# Ostrich eggshell bead diameter in the Holocene: Regional variation with the spread of herding in eastern and southern Africa

**DOI:** 10.1371/journal.pone.0225143

**Published:** 2019-11-27

**Authors:** Jennifer M. Miller, Elizabeth A. Sawchuk

**Affiliations:** 1 Department of Anthropology, University of Alberta, Edmonton, Canada; 2 Department of Archaeology, Max Planck Institute for the Science of Human History, Jena, Germany; 3 Department of Anthropology, Stony Brook University, New York, United States of America; Banaras Hindu University, INDIA

## Abstract

Despite their ubiquity in Holocene African archaeological assemblages, ostrich eggshell (OES) beads are rarely studied in detail. An exception is in southern Africa, where there is a proposed relationship between OES bead diameter and the arrival of herding ~2000 years before present. In 1987, Leon Jacobson first observed that beads from forager sites in Namibia tended to be smaller than those associated with herder sites. Studies examining bead size around the Western Cape have generally confirmed Jacobson’s findings, though the driving forces of the diameter change remain unknown. Since this time, diameter has become an informal way of distinguishing forager and herder assemblages in southern Africa, but no large-scale studies of OES bead variation have been undertaken. Here we present an expanded analysis of Holocene OES bead diameters from southern, and for the first time, eastern Africa. Results reveal distinct patterns in OES bead size over time, reflecting different local dynamics associated with the spread of herding. In southern Africa, OES diameters display low variability and smaller absolute size through time. While larger beads begin to appear <2000 years ago, most beads in our study remained smaller. In contrast, eastern African OES bead diameters are consistently larger over the last 10,000 years and show no appreciable size change with the introduction of herding. Notably, larger beads thought to be associated with herders in southern Africa fall within the range of eastern African beads, indicating a potential connection between these regions in the Late Holocene consistent with genetic findings. Regional differences in bead size are subtle, on the order of millimeters, yet offer a potentially important line of evidence for investigating the spread of herding in sub-Saharan Africa. In order to understand the meaning of these changes, we encourage additional studies of OES bead assemblages and urge researchers to report individual bead diameters, rather than averages by level.

## Introduction

Ostrich eggshell (OES) beads are among the oldest known ornaments made by our species and are often found in archaeological assemblages throughout Africa [1–4]. Although OES beads occur in forager, herder, and farming contexts across the continent, there has been limited study into regional bead variation and the diversity of forms across large distances and time periods. One exception is the Western Cape of southern Africa, where there has been comparatively in-depth research into changing bead diameters over the last 5000 years, and the implications of this for human population dynamics and socioeconomic change (e.g. [5–12]).

Thirty years ago, Leon Jacobson [5,6] proposed a relationship between the diameter of OES beads and the spread of herding into southern Africa. Using a small subset of archaeological sites in Namibia, he observed an increase in bead diameter between foragers and early herders following the introduction of sheep ~2000 years before present (BP). Subsequent research generally confirmed these findings within the western part of southern Africa (e.g. [7,10,13]), and bead diameter has since been informally used a chronological marker at archaeological sites throughout southern Africa. The idea that larger beads are associated with herders has persisted for decades without being systematically tested as new sites and assemblages have been discovered, leading some to caution against its continued use a chronological proxy [14,15]. Furthermore, the bead size hypothesis has been not been evaluated in other parts of the continent, such as eastern Africa, where a similar transition from foraging to mobile herding began some two thousand years earlier.

Here we present an expanded analysis of OES bead diameters from across the Holocene of southern and eastern Africa to explore their diversity on a greater scale. This allows us, for the first time, to test the hypothesis that larger bead diameters are associated with the appearance of herders in two separate geographical areas. We re-evaluate previously published bead diameters from nineteen sites and add datasets from eleven additional sites in Botswana, Kenya, Tanzania, Namibia, and South Africa. By using individual bead diameters rather than averages by level, we increase the dataset on Holocene OES beads from less than 100 data points to >1000. This permits a considerably more nuanced exploration of cultural behaviour as reflected in beads. We recommend future studies adopt this approach in order capture subtle variability within levels and time periods that is masked when bead averages are reported by level.

Beads are often overlooked as analytical units, yet their roles in social expression, identity signaling, and exchange may offer important insights into aspects of the past that are not apparent from other lines of evidence [16–18]. Because they are common artifacts at African archaeological sites spanning >40,000 years ago to recent periods [1,3,4,19], they can be useful in comparisons of cultural practices through time on local, regional, and continental scales [20]. In this article, we demonstrate the value of assessing patterns in OES bead styles across time and space to gain novel perspectives on human interactions, economic transitions, and other social processes evident in the archaeological record of Holocene Africa.

## Background

### The spread of herding to eastern and southern Africa

Pastoralism, or herding domestic animals, was the first form of food production to spread across Africa [21]. Herding began ~8000–7000 BP when sheep and goats of southwest Asian origin, along with cattle with potentially mixed African/southwest Asian ancestry, spread rapidly through the Sahara during the African Humid Period [22–24]. Increasing aridity after ~6000 BP pushed herders out of the desert and along major watersheds to the west and southeast, facilitating the spread of food production into sub-Saharan Africa.

Herding entered eastern Africa through northwestern Kenya ~5000 BP as Lake Turkana receded, exposing new pasturelands [25–27]. The appearance of sheep, goats, and cattle coincided with major cultural changes, including new lithic and ceramic technologies as well as the construction of monumental cemeteries [27,28]. It is unclear whether livestock were introduced primarily through demic diffusion or exchange, but evidence that people engaged in both fishing and herding suggests complex population interactions during this transitional time [28–30].

Cattle did not spread further south for several hundred years, potentially limited by novel livestock disease vectors south of Lake Turkana [31,32]. By ~3300 BP, specialized sheep, goat, and cattle pastoralism became widespread in southern Kenya and northern Tanzania [28,33,34]. Herders entering the South-Central Rift Valley either from the Turkana Basin or elsewhere did not fully displace forager economies, leading to a mosaic of herders and foragers during the Pastoral Neolithic (PN) era [32,35]. Diverse PN cultures thrived for nearly two millennia until the arrival of Iron Age farming ~1600BP, after which time new relationships between foragers, herders, and farmers formed [36,37].

Domesticated caprines entered southern Africa ~2000 BP, although the route(s) and mechanism(s) of this process are contested [15,38–42]. As with eastern Africa, these non-indigenous species must have been introduced via contact with northern populations. This assumption is supported by linguistic [43] and genetic evidence for a connection between eastern and southern Africa [44–49]. What is in question is the timing and extent of contact, the role of migrants in transmitting herding practices, and the degree of cultural diffusion and local adoption.

In southern Africa, competing models for how herding spread must account for the rapid movement of sheep and their low proportions at sites that otherwise yield Later Stone Age (LSA) artifacts. Sheep/goats appeared on the western and southern coasts of South Africa ~2000 BP, virtually simultaneously with their arrival in Namibia ~2200 BP and northern Botswana ~2000 BP [15,38,42,50]. Yet domestic species represent <10% of the fauna at most first millennium AD herding sites [15]. Pottery also spread around this time, although the connection between these processes is debated [15,42]. Cattle appeared several centuries later after 1300 BP, with the exception of a cow horn core dated to 1529–1391 cal BP in Namaqualand [39]. Those seeking to explain this pattern typically fall into three camps: either sheep spread rapidly through down-the-line trade among foragers who independently became “hunters with sheep” [41,51], sheep spread through the demic diffusion of herders ancestrally related to the Khoekhoen [42], or a combination of these processes [38].

Genetic evidence suggests migration and admixture were factors in the spread of herding to both eastern and southern Africa. Based on ancient DNA (aDNA) analysis of 41 foragers and early food producers from Kenya and Tanzania, Prendergast et al. [52] propose a multi-step model for the introduction of herding and farming involving multiple population movements. Pastoral Neolithic herders exhibit ancestry related to both eastern African foragers and northern Africans, with the timing of admixture estimated to have occurred ~4500 BP. This is also when alleles for Lactase Persistence are thought to have entered the region, ostensibly through migration or contact with pastoralists from Ethiopia or Sudan [53,54].

Some eastern Africans herders were seemingly involved in the transmission of herding father south. A ~1200-year-old individual from a herder context at Kasteelberg in South Africa exhibits ancestry related to both southern and eastern African populations, with 40% of her ancestry related to an infant from the PN herder site of Luxmanda in Tanzania [49]. These findings support the hypothesis that a non-Bantu-related population carried eastern African ancestry to southern Africa by 1200 BP. Three ~2000-year-old individuals from forager contexts at Ballito Bay and Doonside, located on the eastern coast of South Africa, do not exhibit eastern African ancestry [48]. However, their genetic distinctiveness from recent southern Africans suggests modern day Khoesan exhibit up to 30% ancestry related to eastern African sources. Admixture is estimated to have occurred >1500 years ago, around the time herding spread, and is consistent with a migration of eastern African pastoralists who admixed with Later Stone Age hunter-gatherers [47,48]. Gene flow is also inferred from Y chromosome (Henn et al. 2008; Batini et al. 2011) and nuclear DNA patterns among contemporary Africans [47,55,56], as well as high frequencies of the most common eastern African allele for Lactase Persistence (14010G>C) among South African Nama herders [45].

Herding’s spread into eastern and southern Africa followed different trajectories in terms of timing, pace, landscapes, and human populations involved. These differences are reflected in distinctive material cultural records, which may extend to differences in OES beads. And yet, these processes were part of a larger movement of non-African domesticates through sub-Saharan Africa. The introduction of sheep, goats, and cattle from the north involved, to varying extents, the same mechanisms of demic diffusion, local adoption, interaction, and innovation as economies shifted and herders and foragers established new relationships. Both regions were then further impacted by the later arrival of Bantu-speaking farmers. The persistence of foragers in both regions across these transitions suggests disparate groups were involved in complex interactions. With this in mind, personal ornaments such as OES beads may provide clues as to how goods and people moved through these landscapes and were influenced by the changes around them.

### Previous studies on OES beads

OES beads are abundant at Holocene sites in eastern Africa but there has been little systematic study of bead size or attributes. A notable recent exception is Tryon et al. [2], where mean diameter by level is reported for beads spanning >40,000 to ~4200 BP at Kisese II Rockshelter in Tanzania. More often, sources report the presence and number of beads associated with Holocene deposits. For example, beads from LSA forager contexts are reported around Lake Turkana [57,58], Lake Victoria [59–61] and Enkapune Ya Muto in the Central Rift Valley [3]. Beads are also found in early Pastoral Neolithic contexts, such as the thousands of OES beads reported from pillar site cemeteries constructed around Lake Turkana by the earliest herders in the region [62]. OES beads can also be found, though in lower quantities, in PN contexts further south at sites such as Gogo Falls [61], Keringet Cave [63], Porcupine Cave [64], Gambles Cave II [65], Lukenya Hill (GvJm 22) [66], Lion Hill Cave, Akira, and Salasun [67], and Luxmanda [68]. There may be more examples, but most sources focus on other types of PN ornaments such as stone and seed beads [69].

Considerably more attention has been paid to OES bead variation in Holocene southern Africa. Jacobson [5,6] suggested that OES bead diameters vary over time based on observations by W.E. Wendt and P. Wiessner about archaeological surface scatters, and ethnographic work among the Kalahari San. To test the idea, Jacobson analyzed 18 bead assemblages from seven sites, divided into three types: pre-herding foragers with microlithic assemblages but no pottery; foragers or transitional early herders with small amounts of pottery; and herders with few stone artifacts but with abundant pottery. He found the greatest differences in mean bead diameter between pre-herding foragers and herder sites, with no beads larger than 7.5 mm found in any of the foraging sites, although smaller beads were found in both. He therefore suggested that sites with larger beads were likely to be later, though the absence of larger beads did not necessarily signify an earlier site.

Bead diameter was soon adopted by researchers looking for ways to differentiate forager from herder archaeological assemblages and to distinguish herders from foragers on their periphery who may have used livestock as part of their seasonal cycle. By looking at bead size change through time at sites in the Western Cape, Smith et al. [7] observed a clear and consistent distinction between the sites of Witklip (associated with foragers) and Kasteelberg A and B (associated with herders). Combined with other material cultural evidence from lithic toolkits, faunal assemblages, and ceramic indices, they used this to argue for maintenance of distinct cultural groups of foragers and herders after the arrival of herding. This implied bead size could be used as an indicator of cultural identity, and not just time.

Subsequent studies sought to test changes in bead diameter in other parts of southern Africa [8–13,70,71]. While most validated the increase in bead size over time, some researchers questioned the association with herders [14,72]. Sadr and colleagues [10] re-evaluated the assemblages studied by Smith et al. [7] and argued that differences in bead size were driven by cultural evolution in bead-making as opposed to the preferences of distinct groups. Orton et al. [9] found smaller beads at Rooiwal Midden that fell into forager size ranges, yet were dated to later herder time periods and associated with pottery. This echoes Jacobson’s caveat that an absence of larger beads does not necessarily indicate older assemblages. Kandel and Conard [8] confirmed that smaller beads were associated with pre-pottery forager assemblages, however, noted that one larger bead (7.4 mm) was directly dated to the pre-herding period (3907–4087 cal BP), and cautioned that the smaller vs. larger bead dichotomy may not be universal. A similar result was produced by Orton and Compton [73] who directly dated a 6.0 mm bead to 6913–7162 cal BP, well before herding spread to southern Africa. These studies highlight variability within forager and herder assemblages; what is considered small in some cases is average in others. The nature of changes in bead size seems to be regionally variable, but Jacobson’s observation about increase over time has been largely validated.

**Fig 1:**
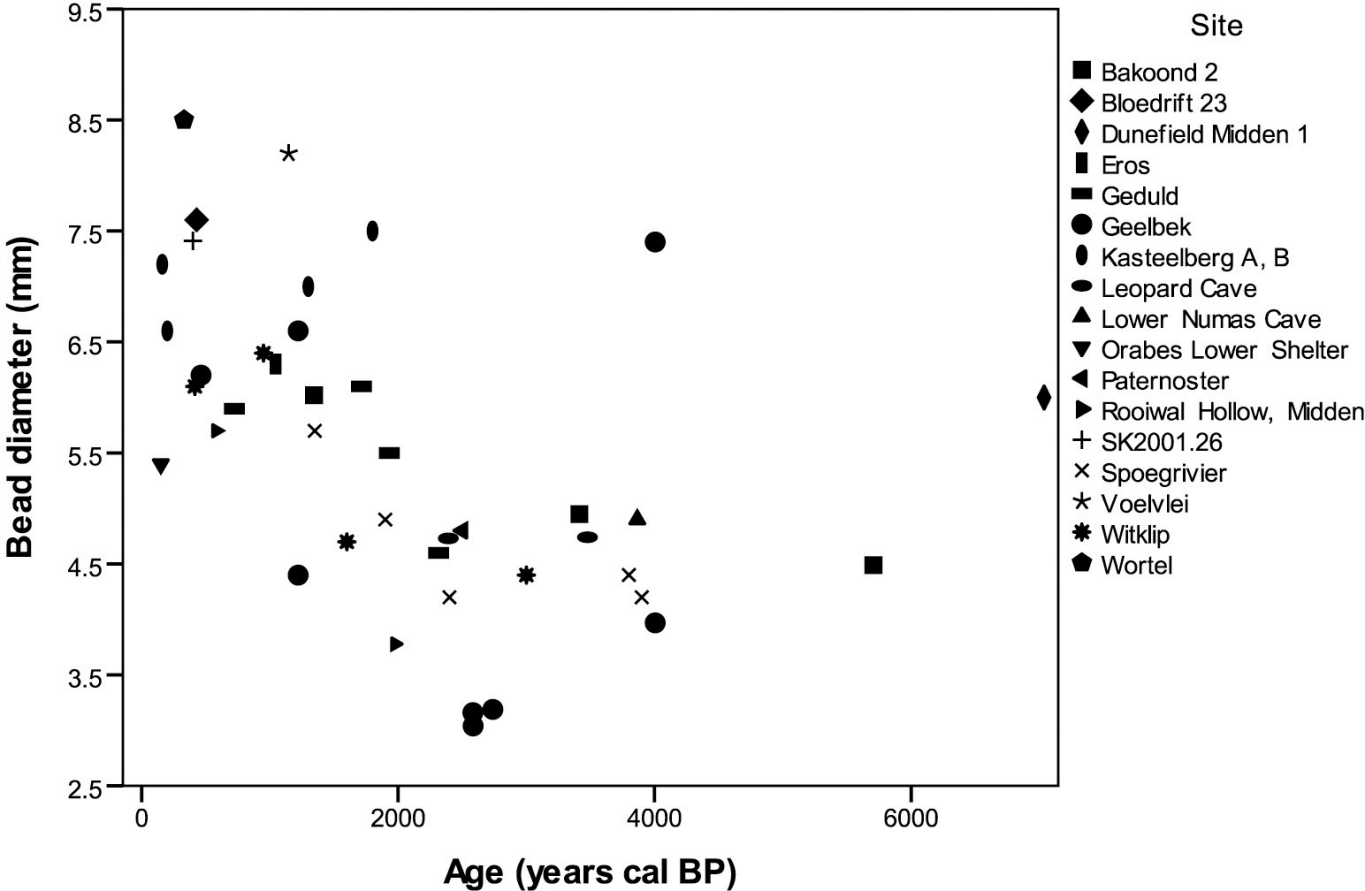
Previously published OES bead diameter data for southern Africa. Produced using data from [5–10,12,73–76].

Despite an emerging picture of complexity across time and space, there has been limited consideration of results between studies. When these early bead data are plotted on a single graph, the inverse relationship between bead size and time is evident (Fig 1). However, most of these points represent average diameters by excavation level or strata, obscuring variation among individual beads. A few larger beads in an otherwise small-diameter assemblage may skew the results. Grouping by excavation level also renders it difficult to compare assemblages between sites. Furthermore, it remains unclear whether an increase in bead size occurred in other parts of the continent where herding was introduced, such as eastern Africa where beads have a similarly long history [1,33]. By combining published and new data on bead diameter change through time using a finer-grained approach, we revisit the question of bead diameter change in southern Africa and compare results to similar processes in eastern Africa. In doing so, we hope to contribute another line of evidence to debates on how herding spread, and the human interactions underlying these important transitions.

## Materials and methods

**Fig 2:**
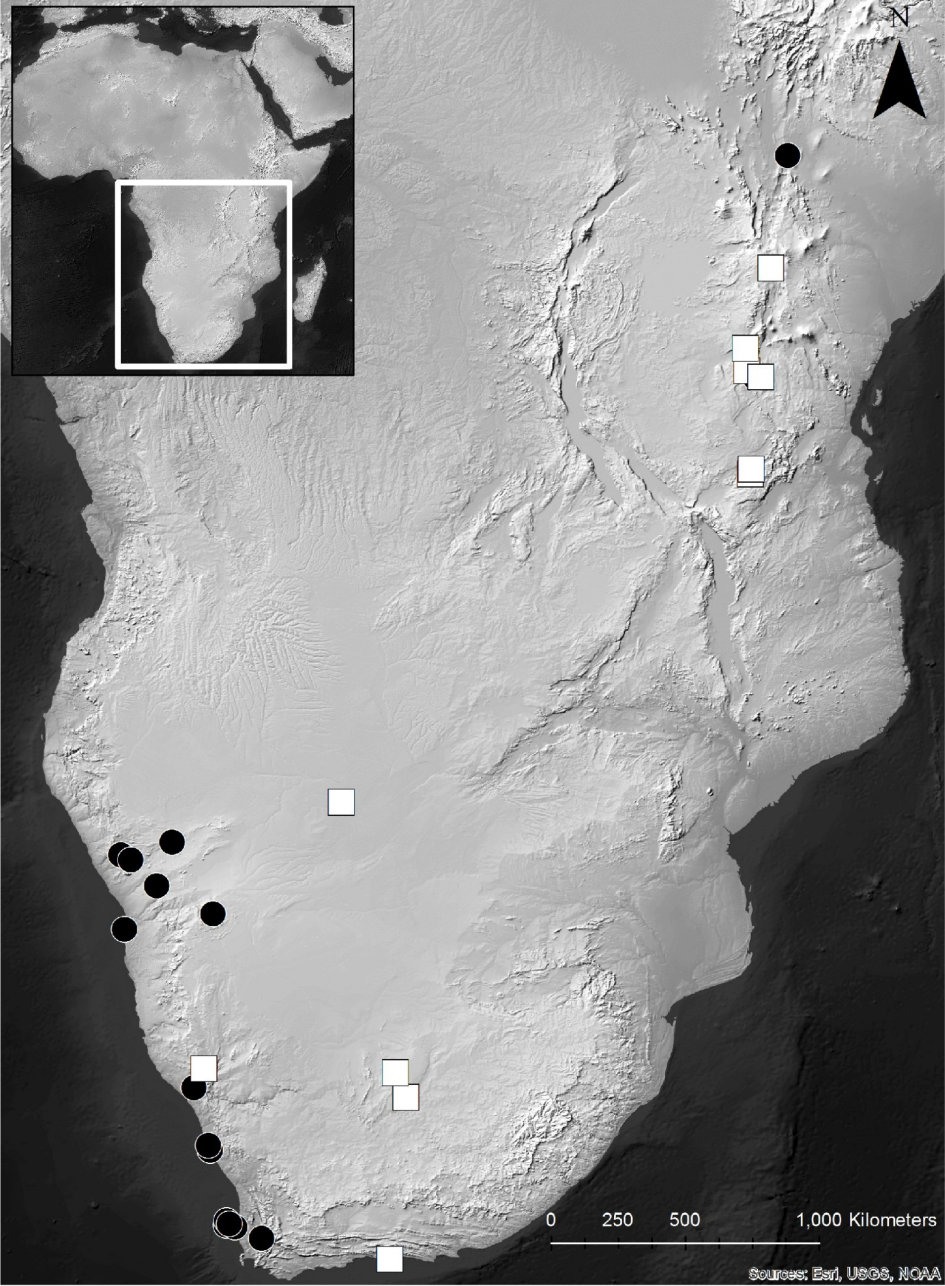
Map of sub-Saharan Africa showing locations of sites used in this study. New data shown with white squares, previously published data with black circles; basemap courtesy of Natural Earth (naturalearthdata.com).

We measured, or compiled published measurements, for beads from Holocene deposits from eight sites in eastern and 22 sites in southern Africa (n = 1200) (Fig 2, Table 1). Dates used are based on associated radiocarbon dates for levels in which the beads were found, or bracketing dates for surrounding levels. These indirect dates have associated margins of error and should be interpreted with the appropriate caution. Three beads included in this study have direct radiocarbon dates (Dunefield Midden 1 [73], Geelbek Loop [8], and Magubike Rockshelter [1]). Wherever possible, published radiocarbon ages were calibrated using the Intcal13 dataset [77] and graphed with the median calibrated age. We selected an arbitrary maximum age of 10,000 years BP for beads to include in this study, which excluded many beads including those from Bushman Rockshelter, which date to 10,930 cal BP [78]. Our data include completed beads (Stage 7a), as well as completed but broken beads with >50% of the diameter preserved (Stage 7b) (stages per [11]).

**Table 1:** List of sites with OES diameters used in this study.

Site	Country	# of data points^a^	Reference
White Paintings Shelter	Botswana	3	^b^
Apollo 11 Cave	Namibia	6	^b^
Eros	Namibia	1	[5]
Geduld	Namibia	4	[5]
Leopard Cave	Namibia	2	[74]
Lower Numas Cave	Namibia	1	[5]
Orabes Lower Shelter	Namibia	1	[5]
Wortel	Namibia	1	[5]
Bakoond 2	South Africa	3	[75]
Bloeddrift 23	South Africa	1	[12]
Dikbosch 1 Rockshelter	South Africa	3	^b^
Dunefield Midden 1	South Africa	1	[73]
Geelbek Dunes	South Africa	8	[8]
Kasteelberg A,B	South Africa	4	[10]
Nelson Bay Cave	South Africa	514	^b^
Paternoster	South Africa	1	[7]
Rooiwal Hollow,Midden	South Africa	2	[9]
SK2001.26	South Africa	1	[9,79]
Spoegrivier Cave	South Africa	5	[76]
Voëlvlei	South Africa	1	[7]
Witklip	South Africa	4	[7]
Wonderwerk Cave	South Africa	333	^b^
Enkapune Ya Muto	Kenya	66	^b^
Lowasera	Kenya	1	[60]
Daumboy 3 Rockshelter	Tanzania	2	^b^
Kisese II Rockshelter	Tanzania	103	^b^
Luxmanda	Tanzania	2	[68]
Magubike Rockshelter	Tanzania	19	^b^
Mlambalasi Rockshelter	Tanzania	19	[80], ^b^
Mumba Rockshelter	Tanzania	88	^b^
**Total**		**1200**	

^a^‘Data point’ refers to a single point with known diameter and age estimate, and may represent a reported mean of several beads grouped by excavation level.

^b^Diameter data originates from this study.

Previous work by Yates [81] advocated for the use of the maximum diameter measurement as a way to reduce the effects of use wear on bead size, however this assumes that all beads are intended to be circular. Many finished beads are oval, oblong, blocky, or only roughly circular. Intuitively, prolonged use wear will reduce the diameter of OES beads, however these effects have not been formally studied. In order to account for non-circular beads, we took multiple measurements recording minimum and maximum values, and used these to generate an average diameter for analysis.

For most of the new data, bead diameter was measured manually (by JM) using standard digital calipers. A few collections (Daumboy 3, Magubike, Mlambalasi, and Mumba Rockshelters) were studied at the University of Alberta while exported on-loan by their respective excavators, while other collections (White Paintings Shelter, Apollo 11 Cave, Dikbosch 1 Rockshelter, Nelson Bay Cave, and Wonderwerk Cave) were studied in the southern African museums in which they are curated (refer to S1 File for details). Measurements for beads from Enkapane Ya Muto were recorded from high resolution digital microscope photos taken from directly overhead (by Dr. Phillip Slater, supervised by Dr. Stanley Ambrose), with measurements processed in ImageJ [82] by JM. Beads from Kisese II were measured by Dr. Christian Tryon, and the individual diameters used to generate the level averages in Tryon et al. [2] are included here as separate entries.

Data points for sites originating from this study tend to be more numerous than for sites with previously published data, because individual beads are being graphed. Data points for published sites (where diameter was reported as mean by level) typically reflect the average of measurements for multiple beads that can only be entered as a single point. All data used here are available in the S1 Table.

This is not an exhaustive list of OES beads from eastern and southern African Holocene archaeological sites. We chose a subsection of sites with deposits spanning the spread of herding to these regions, and that had beads with published data or those that were accessible for study, with known provenience and associated radiocarbon dates. We hope that this model will be expanded in the future to incorporate additional datasets.

For this study we use the terms ‘larger’ and ‘smaller’ only as relative descriptors within these datasets, and not to refer to absolute size cutoffs. There have been varying definitions of ‘large’ and ‘small’ beads in the literature in the context of their relationships to hunters and herders. Jacobson’s [5,6] data indicated that 7.5 mm was a boundary, with beads larger than that threshold post-dating the spread of herding. Orton [70,71] proposed size classes based on measurements, calling all beads ≤5.00 mm small and those 6.01–7.50 mm large. Since we advocate for the use of individual bead data points, there is no need to translate the diameters into categories, or to standardize the description of bead sizes.

## Results

**Fig 3:**
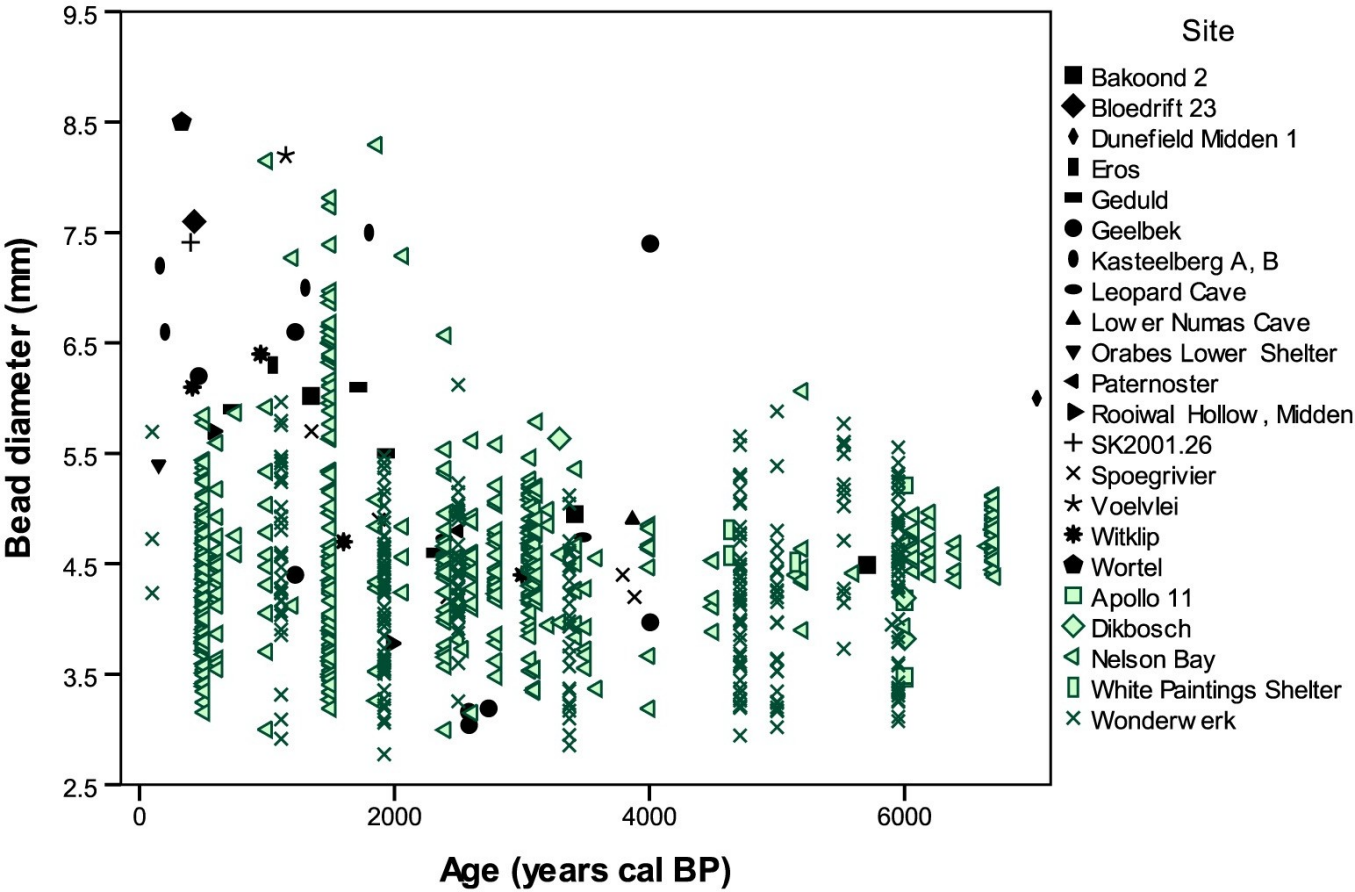
OES bead diameters from southern Africa. New data shown in green, previously published data in black.[83,84].

Graphing the new individual bead diameters in addition to the previously published measurements provides a more nuanced perspective of the southern African data (Fig 3). There is relatively low variability in bead diameters in southern African from 10,000–2000 BP, and their overall size is smaller (mean = 4.43 mm, range 2.86–7.40 mm, n = 544). The benefit of plotting individual beads, as opposed to summary data, becomes apparent when examining diameters from 2000 BP to present. With this refined perspective, smaller beads still make up most of the examples we examined, despite the appearance of some larger beads (mean = 4.63 mm, range 2.78–8.50 mm, n = 356) in younger periods.

Larger beads do not appear in greater numbers until <2000 BP. In fact, all recorded beads older than 2500 BP (n = 506) are 6 mm or smaller, with only three exceptions (Dunefield Midden 1, Nelson Bay Cave, and Wonderwerk Cave). The direct date on the Dunefield Midden bead renders its age reliable, however the other two cases should be regarded cautiously for several reasons. The Nelson Bay Cave assemblage was excavated from 1964–1979 and went through changes in layer naming systems as well as a transition from imperial to metric measurements. Wonderwork Cave has been excavated intermittently since the 1940s and also went through several layer naming conventions, summarized in [85]. It is thus unclear whether these are early examples of larger beads penetrating southern Africa, or whether their stratigraphic attributions—by which their ages are estimated—are inaccurate. Overall, the data suggest most beads in the southern African sample remained smaller throughout the Holocene, despite the addition larger beads in later time periods.

A different pattern emerges for eastern Africa. Compared to southern African diameters, bead sizes in eastern Africa are greater over the last 10,000 years, and do not appear to change after the introduction of herding. Average bead diameter from assemblages >4000 BP is 6.15 mm, with a range of 4.39–9.15 mm (n = 188). Beads younger than 4000 BP have a mean of 6.65 mm with a range of 4.73–14.49 mm (n = 112). The 14.49 mm specimen is an unusually large outlier, and the next largest beads are <10 mm. Although diameter changes slightly through time, overall bead size remains variable.

**Fig 4:**
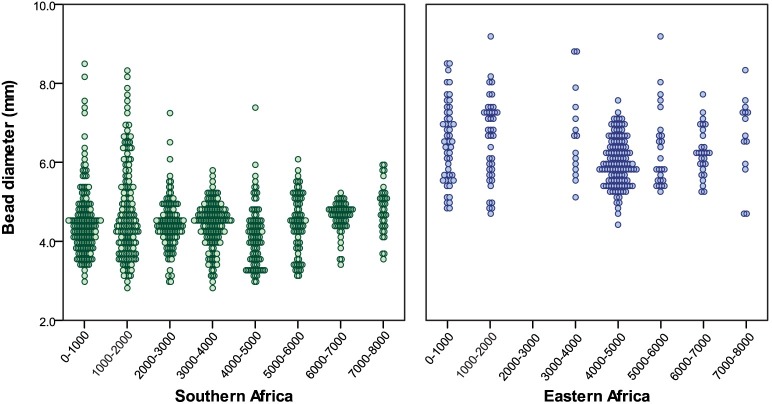
OES bead diameters from southern and eastern Africa. Southern data shown in green, eastern data in blue. One eastern African outlier (14.49 mm) is not shown.

**Fig 5:**
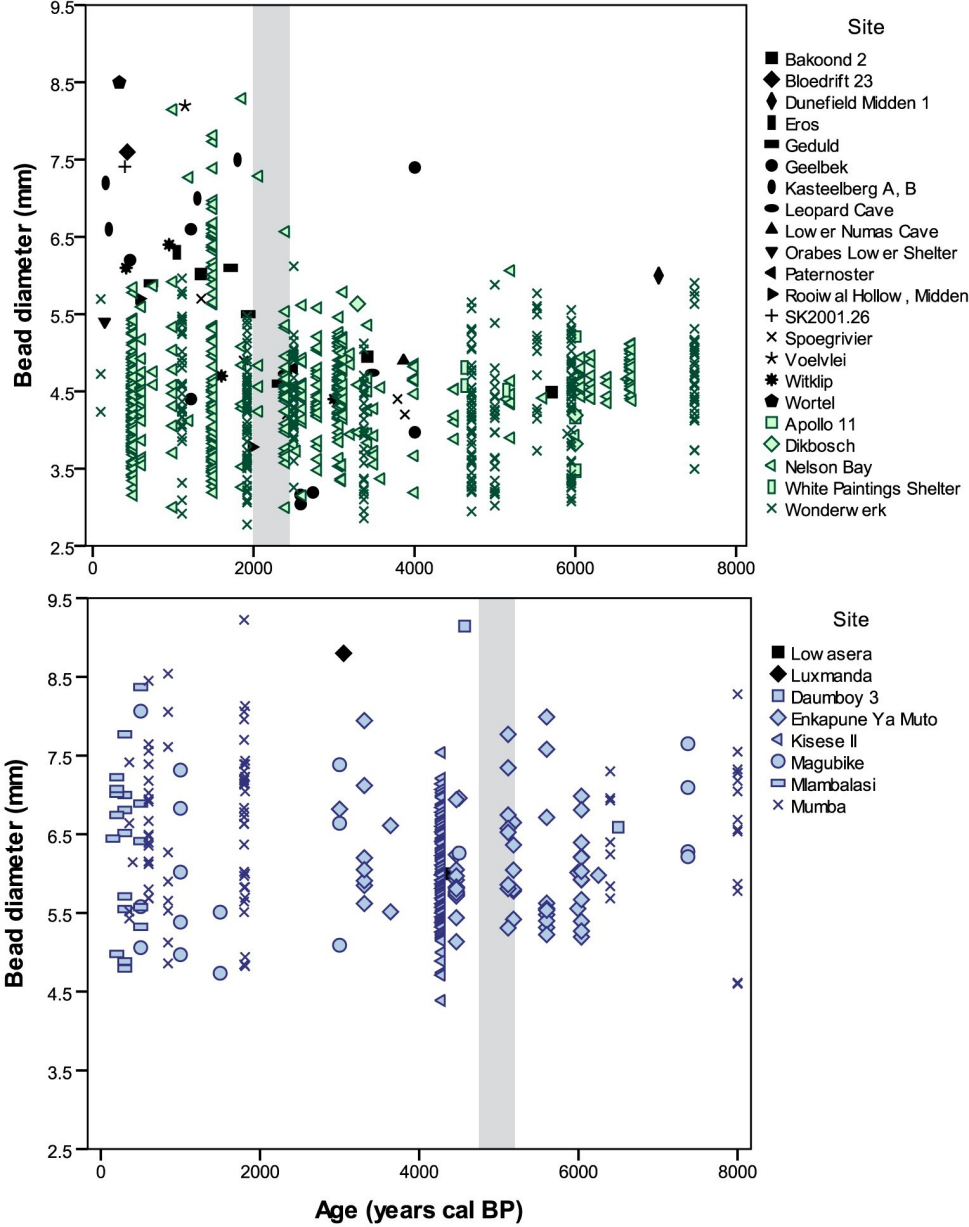
Scatter plots of OES bead diameters from southern and eastern Africa. New southern data shown in green, new eastern data in blue, previously published data in black. Grey bars represent the introduction of herding into each region. Two eastern African outliers (14.49 and 10.07 mm) are not shown.

**Fig 6:**
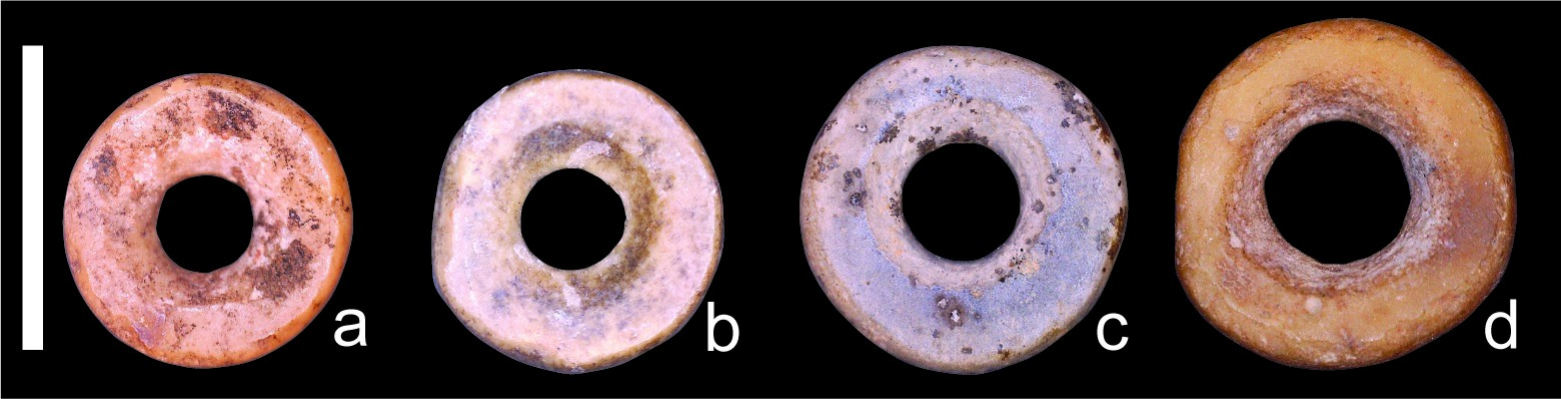
Examples of OES beads showing subtle size differences between regions. Scale bar = 5 mm; (a) Nelson Bay Cave, South Africa; (b) Wonderwerk Cave, South Africa; (c) Magubike Rockshelter, Tanzania; (d) Daumboy 3 Rockshelter, Tanzania.

Comparing eastern and southern African datasets reveals different regional patterns (Fig 4). The eastern African beads are consistently larger both before and after the initial introduction of herding to the region, barely overlapping with the upper reaches of southern African beads. There is also no apparent trend in bead diameter size through time. In southern Africa, only beads younger than 2000 BP overlap with eastern African values, thanks to the addition of a few larger examples. Even so, most of the young southern African beads remain smaller. When individual bead data from eastern and southern Africa are plotted together, the greatest overlap between regions occurs <2500 BP (Fig 5). These differences between regions appear striking, but only vary on the order of millimeters. Without a comparative collection or familiarity with OES bead variation, these differences would not be apparent, and would likely go unrecognized by researchers working in separate regions (Fig 6).

## Discussion

### Bead patterns differ with the spread of herding in eastern and southern Africa

Patterns of OES bead diameter in eastern and southern Africa are patently different. Both regions acquired herding through contact with populations from the north, and both regions had prior, long-standing OES bead traditions. Yet regional and temporal patterns in OES bead diameters reflect this transition in unique ways. This begs the question of what beads can tell us about the introduction of herding to different parts of the continent.

In eastern Africa, OES beads size did not appear to change with the arrival of herding despite other shifts in material culture. This may be, at least partly, because livestock were adopted by local foragers who retained their bead-making traditions. It is also possible that migrants entering eastern Africa possessed similar bead traditions, or adopted local traditions soon after arriving through contact and/or integration with foragers. These possibilities are not mutually exclusive, and multiple factors likely contributed to herding’s spread. However, using beads to distinguish between different trajectories requires further research on pastoralist artifact assemblages farther north. OES beads have been reported from numerous sites in the Nile Valley including Gebel Ramlah [86], el Barga [87], el-Kadruka [88], Esh Shaheinab [89]; R12 [90], el-Kadada [91] and el Geili [92]—all of which predate herding’s spread into eastern Africa—but virtually no information has been published on beads or their diameters. Additionally, although pillar site cemeteries around Lake Turkana have yielded thousands of OES beads, these have also yet to be systematically studied or measured. However, the sheer volume of OES beads at the pillar sites imply they were important for identity signaling and exchange among early herders and likely foragers interacting along this frontier [27,28,62].

In eastern African sites where beads are known from both pre- and post-herding contexts, there is virtually no change in bead diameter over time. Although it is unclear for many of the measured sites whether beads were associated with herders or foragers, after ~3000 BP these groups were likely in contact, or at least aware of one another. If the development of PN traditions in the Rift Valley disrupted pre-existing social networks, this is not reflected in OES bead diameters.

In southern Africa, there is a detectable change in bead diameters that appears to correlate with herding’s spread. Although the exact timing is disputed, zooarchaeological data support an initial entrance of sheep/goats around 2000 BP, exactly when a subset of >6.5 mm OES beads appear. The introduction of new bead sizes seems coeval with the arrival of herding, but larger beads did not replace existing traditions or become dominant in the assemblages we sampled. This is consistent with other evidence of cultural continuity in the archaeological record [15,40]. Two interpretations follow: either the arrival of livestock stimulated a local change in bead traditions, or more likely, contact with foreign groups introduced new bead styles into southern Africa along with domesticates. Whether this process entailed infiltration of the bead-makers, or simply the movement of beads, is unknown. Continuity in smaller bead assemblages suggests either domesticates and foreign bead styles were acquired through exchange and adopted locally, or that migrants accompanying livestock integrated into local communities. Although we cannot reconstruct the underlying social processes, timing suggests the arrival of livestock meaningfully shifted social practices, networks, or both.

These results also lend support for Jacobson’s [5,6] original use of larger OES beads as a chronological indicator. Because beads >6.5 mm only appeared in large numbers over the last 2000 years, their presence can be used with other lines of evidence to tentatively identify sites post-dating the arrival of herding in southern Africa. Bead size alone, however, should not be used to ascribe an assemblage or archaeological site to herders in southern (or eastern) Africa, as we do not yet understand how beads transferred between hunters and foragers, or the relationship between these groups in antiquity. Likewise, because smaller beads dominate throughout time, the absence of larger beads should not be used to infer a pre-herder assemblage, just as Jacobson warned. Although bead size should not be used as a sole chronological indicator, these results support some association between larger beads and occupations younger than ~2000 BP, in southern Africa.

Comparisons of eastern and southern African OES bead assemblages support other lines of evidence showing different trajectories in the introduction of livestock and herding. Although both scenarios likely involved population interaction and exchange, there is only an observable change in bead styles in southern Africa. Examining these different patterns more closely in conjunction with other archaeological data may reveal what interactions looked like and unique pathways to food production on the African continent. Since OES beads were used by foragers for more than 40,000 years in both regions, change—or lack thereof—in bead styles is informative about how foragers responded, coped, and mediated cultural contact and economic change. Given the ubiquity of OES beads at African archaeological sites bridging this transition, beads constitute important evidence for how the arrival of herding impacted diverse human landscapes.

### A new way to explore interregional contact?

Patterns in OES beads may also provide an independent line of evidence connecting eastern and southern Africa in the Late Holocene. The hypothesis that sheep and goats were introduced to southern Africa by migrants who were the ancestors of Khoekhoen herders, has a long history of debate in southern African archaeology (summaries in [42,93,94]). Although studies have focused on the extent to which herding was introduced via migration or cultural diffusion, the origins of northern populations transmitting livestock also remains in question. Size similarities between larger OES beads in eastern African and later southern African contexts may point to a connection with the herding intensive PN cultures of the South-Central Rift Valley.

Multiple genetic studies now document gene flow from eastern to southern Africa around the time herding spread, providing unequivocal evidence of contact between these regions consistent with other lines of archaeological evidence. This is especially evident in the shared ancestry of girls buried at Luxmanda, Tanzania and Kasteelberg, South Africa [49]. What remains in question is the nature of this connection, and whether migration and admixture played a role in the initial spread of herding or occurred later as interregional population dynamics evolved. Importantly, the scale of admixture was small and inconsistent with a major migration or population replacement [44,46,47]. Rather, small groups from eastern Africa, or who were in contact with eastern Africans, appear to have interacted and potentially integrated with southern African foragers. This is supported by the genetic relatedness of Khoe and San groups despite evidence of some eastern African admixture, which Uren et al. [95] use to argue that herding primarily spread through cultural diffusion. Additional sequences from ancient southern African foragers and herders may help refine the timing and extent of contact and admixture.

Patterns in southern African OES beads seem to parallel genetic evidence for interregional contact, but add the perspective of cultural identity rather than genetic ancestry. OES beads similar in diameter to those from eastern Africa appeared around the same time as livestock, but did not replace existing smaller bead traditions. One explanation for this is that small numbers of migrant herders brought finished beads and/or produced beads using eastern African stylistic norms. These items then entered local knowledge and exchange networks along with livestock and herding practices. It is also noteworthy that both genetic and bead evidence suggests this was a unidirectional process—neither smaller beads, nor southern African genetic signatures, appear to flow back to eastern Africa. Whether beads capture an additional link between eastern and southern African populations around 2000 BP is an intriguing possibility that should be tested through future research.

### Individual bead measurements versus summarized data

A final point that emerges from this research is the importance of reporting individual bead diameters instead of a single averaged calculation by excavation or stratigraphic level. Comparing Figs 1 and 3 demonstrates the masking effect of using averages by level. Previous arguments for increasing bead size through time in southern Africa have been skewed by the addition of larger beads toward the end of this sequence, obscuring the continuity of smaller bead traditions. When the data points are plotted individually, the picture that emerges is one where people incorporated new styles into existing mosaics as opposed to replacing earlier traditions. We suggest that future studies present individual bead diameters, and publish the raw measurements, to facilitate more effective research on bead variation in datasets from across the African continent and beyond.

## Conclusions

Beads constitute a crucial component of African archaeological assemblages, but their usefulness for exploring transitions in the past has been largely overlooked. Reassessment of published and new data on bead size diameter supports Jacobson’s [5,6] observations that bead size increases after the introduction of herding to southernmost Africa. However, the situation is more complex than an association between larger beads and herders. Further, analysis of bead size change with herding’s arrival in eastern Africa reveals a different pattern, one of consistently larger and more variable beads. These results point toward distinct trajectories in the spread of herding to different parts of the continent. Similarities between eastern African beads and the larger beads found in southern African herding contexts may constitute independent evidence for a link between these regions in the Late Holocene consistent with emerging genetic data.

These findings should now be evaluated against other lines of archaeological evidence and tested with additional datasets from these regions and other parts of the continent. Future research should also investigate related questions on bead origins, manufacture, and use, e.g., the effects of taphonomic processes and wear on bead diameter [20,96], regional variation in shell structure and related workability [97], and the extent to which beads and raw materials were moving within and between regions using isotopic analysis. Prior work on manufacture pathways [8,11] should be expanded to examine variation among bead production techniques in greater detail. Finally, additional direct dates on beads would help refine chronological patterns across the continent. Revisiting questions about OES beads in Holocene Africa suggests these small but important artifacts reveal more than previously anticipated, and that they deserve greater attention in our efforts to reconstruct the past.

## Supporting information

S1 File Locations of beads analyzed in this study. (PDF)

S1 Table Bead diameter measurements and age estimates for all sites. Entries 1–44 are published diameters, 45–1220 are new data. For new data, reference refers to publication of the ages. All Kisese II diameters were measured by Dr. Christian Tryon. Wherever possible, published radiocarbon ages were calibrated using the Intcal13 dataset [77] and graphed using the median calibrated age.
(XLSX)

S1 References List of references from S1 Table. (DOCX)
