# Supplementary material for: Ostrich eggshell bead diameter in the Holocene: Regional variation with the spread of herding in eastern and southern Africa
Source: PLoS One. 2019 Nov 27;14(11):e0225143. doi: 10.1371/journal.pone.0225143 (PMC6880992; doi:10.1371/journal.pone.0225143)
Supplement: S1 File — (PDF) [file pone.0225143.s001.pdf]

S1 File - All new data presented in this study was collected either in person, or via other researchers, with appropriate permissions.

Apollo 11 Cave - Studied in Windhoek, Namibia, in 2014, at the National Museum of Namibia. Research permission granted by Dr. Emma Imalwa, Curator of Archaeology at the National Museum of Namibia. All studied material stayed in the National Museum of Namibia, Windhoek.

Daumboy 3 - Studied in Edmonton, Canada, in 2017, while exported on-loan to Dr. Mary Prendergast. Research permission granted by the Tanzanian Commission on Science and Technology (COSTECH) to Dr. Prendergast (2012-167-NA-2012-50), with fieldwork and excavation permission from the Division of Antiquities, Ministry of Natural Resources and Tourism (Government of Tanzania). All studied material is now housed at the National Museum of Tanzania, Dar es Salaam.

Dikbosch 1 - Studied in Kimberley, South Africa, in 2014, at the McGregor Museum. Research permission granted by Dr. David Morris, Head of the Archaeology Department at the McGregor Museum, to Jennifer Miller. All studied material remained in the McGregor Museum, Kimberley.

Enkapune Ya Muto – Studied by Drs. Phil Slater and Stanley Ambrose. Digital microscope photographs taken by Dr. Slater were shared with Jennifer Miller, and data obtained from the photos was approved for analysis. The photos themselves are not approved to be shared.

Kisese II Rockshelter – Studied by Dr. Christian Tryon, summary results published in 2018, raw data shared with Jennifer Miller and approved for use in this manuscript.

Magubike and Mlambalasi - Studied in Edmonton, Canada, between 2010 and 2018, while exported on-loan to Dr. Pamela Willoughby. Research permission granted by the Tanzanian Commission on Science and Technology (COSTECH) to Dr. Willoughby (2018-106-NA-2003-02; 2016-293-ER-2005-154; 2012-245-ER-25005-154; 2010-112-ER-2005-154; 2008-203-ER-2005-154), and the Division of Antiquities, Ministry of Natural Resources and Tourism (Government of Tanzania). All studied material is now housed at the National Museum of Tanzania, Dar es Salaam.

Mumba - Studied in Edmonton, Canada, in 2017, while exported on-loan to Dr. Mary Prendergast. Research permission granted by the Tanzanian Commission on Science and Technology (COSTECH) to Dr. Prendergast (2005-166-CC-2003-96), with fieldwork and excavation permission from the Division of Antiquities, Ministry of Natural Resources and Tourism (Government of Tanzania). All studied material is now housed at the National Museum of Tanzania, Dar es Salaam.

Nelsons Bay Cave - Studied in Cape Town, South Africa, in 2014, at the Iziko Museums of South Africa. Research permission granted by the Iziko Museums of South Africa to Jennifer Miller. All studied material remained in the Iziko Museum, Cape Town.

White Paintings Shelter - Studied in Gaborone, Botswana, in 2014, at the National Museum and Monuments in Gaborone. Research permission granted by the Ministry of Environment, Wildlife and Tourism (reference EWT 8/36/4 XXVI 33), on behalf of the Government of Botswana, to Jennifer Miller. All studied material remained in the National Museum, Gaborone.

Wonderwerk Cave - Studied in Kimberley, South Africa, in 2014, at the McGregor Museum. Research permission granted by Dr. David Morris, Head of the Archaeology Department at the McGregor Museum,

and by Dr. Michael Chazan, to Jennifer Miller. All studied material remained in the McGregor Museum, Kimberley.
