# Supplementary material for: Ostrich eggshell bead diameter in the Holocene: Regional variation with the spread of herding in eastern and southern Africa
Source: PLoS One. 2019 Nov 27;14(11):e0225143. doi: 10.1371/journal.pone.0225143 (PMC6880992; doi:10.1371/journal.pone.0225143)
Supplement: S1 References — (DOCX) [file pone.0225143.s003.docx]

**S1 Table References**

1. Jacobson L. The size variability of ostrich eggshell beads from central Namibia and its relevance as a stylistic and temporal marker. South Afr Archaeol Bull. 1987; 55–58. doi:10.2307/3887775

2. Jacobson L. More on ostrich eggshell bead size variability: The geduld early herder assemblage. South Afr Archaeol Bull. 1987; 174–175. doi:10.2307/3888746

3. Pleurdeau D, Imalwa E, Détroit F, Lesur J, Veldman A, Bahain JJ, et al. “Of sheep and men”: Earliest direct evidence of caprine domestication in southern Africa at Leopard Cave (Erongo, Namibia). PLoS ONE. 2012;7: e40340. doi:10.1371/journal.pone.0040340

4. Orton J. Hunters or herders?: Evidence from the cultural assemblages at Bakoond, Western Cape, South Africa. Farming. 2009; 1–16. doi:10.3828/bfarm.2009.4.1

5. Smith AB, Halkett D, Hart T, Mutti B. Spatial patterning, cultural identity and site integrity on open sites: Evidence from Bloeddrift 23, a pre-Colonial herder camp in the Richtersveld, Northern Cape Province, South Africa. South Afr Archaeol Bull. 2001;56: 23–33. doi:10.2307/3889025

6. Orton J, Compton JS. A reworked mid-Holocene lithic assemblage at Dunefield Midden 1, Elands Bay, South Africa. South Afr Archaeol Bull. 2006; 7.

7. Kandel AW, Conard NJ. Production sequences of ostrich eggshell beads and settlement dynamics in the Geelbek Dunes of the Western Cape, South Africa. J Archaeol Sci. 2005;32: 1711–1721. doi:10.1016/j.jas.2005.05.010

8. Sadr K, Smith A, Plug I, Orton J, Mutti B. Herders and foragers on Kasteelberg: Interim report of excavations 1999-2002. South Afr Archaeol Bull. 2003;58: 27–32. doi:10.2307/3889154

9. Smith AB, Sadr K, Gribble J, Yates R. Excavations in the South-Western Cape, South Africa, and the archaeological identity of prehistoric hunter-gatherers within the last 2000 years. South Afr Archaeol Bull. 1991;46: 71–91. doi:10.2307/3889087

10. Orton J, Hart T, Halkett D. Shell middens in Namaqualand: Two Later Stone Age sites at Rooiwalbaai, Northern Cape Province, South Africa. South Afr Archaeol Bull. 2005; 24–32. doi:https://www.jstor.org/stable/3889045

11. Webley L. The re-excavation of Spoegrivier Cave on the West Coast of South Africa. Ann East Cape Mus. 2002;2: 19–49.

12. Phillipson DW. Lowasera. Azania Archaeol Res Afr. 1977;12: 1–32. doi:10.1080/00672707709511245

13. Langley MC, Prendergast ME, Grillo KM. Organic technology in the Pastoral Neolithic: Osseous and eggshell artefacts from Luxmanda, Tanzania. Archaeol Anthropol Sci. 2017;11: 1–14. doi:10.1007/s12520-017-0528-z

14. Robbins LH, Murphy ML, Brook GA, Ivester AH, Campbell AC, Klein RG, et al. Archaeology, palaeoenvironment, and chronology of the Tsodilo Hills White Paintings Rock Shelter, northwest Kalahari Desert, Botswana. J Archaeol Sci. 2000;27: 1085–1113.

15. Ambrose SH. Chronology of the Later Stone Age and food production in East Africa. J Archaeol Sci. 1998;25: 377–392. doi:10.1006/jasc.1997.0277

16. Murray-Wallace CV, Richter J, Vogelsang R. Aminostratigraphy and taphonomy of ostrich eggshell in the sedimentary infill of Apollo 11 Rockshelter, Namibia. J Archaeol Sci Rep. 2015;4: 143–151. doi:10.1016/j.jasrep.2015.09.006

17. Vogelsang R, Richter J, Jacobs Z, Eichhorn B, Linseele V, Roberts RG. New excavations of Middle Stone Age deposits at Apollo 11 Rockshelter, Namibia: Stratigraphy, archaeology, chronology and past environments. J Afr Archaeol. 2010;8: 185–218. doi:10.3213/1612-1651-10170

18. Humphreys AJB. A preliminary report on test excavations at Dikbosch Shelter I, Herbert District, Northern Cape. South Afr Archaeol Bull. 1974;29: 115. doi:10.2307/3888337

19. Inskeep RR. Nelson Bay Cave, Cape Province, South Africa: The Holocene Levels. England: British Archaeological Reports; 1987. doi:10.2307/3887623

20. Inskeep RR, Vogel JC. Radiocarbon dates from the Holocene levels at Nelson Bay Cave, and an interim report on their associations. South Afr Archaeol Bull. 1985;40: 103. doi:10.2307/3888456

21. Lee-Thorp JA, Ecker M. Holocene environmental change at Wonderwerk Cave, South Africa: Insights from stable light isotopes in ostrich eggshell. Afr Archaeol Rev. 2015;32: 793–811. doi:10.1007/s10437-015-9202-y

22. Ecker M, Botha-Brink J, Lee-Thorp JA, Piuz A, Horwitz LK. Ostrich eggshell as a source of palaeoenvironmental information in the arid interior of South Africa: A case study from Wonderwerk Cave. Changing Climates, Ecosystems and Environments Within Arid Southern Africa and Adjoining Region. Boca Raton: CRC Press; 2015. doi:10.1201/b19410

23. Prendergast ME, Mabulla AZP, Grillo KM, Broderick LG, Seitsonen O, Gidna AO, et al. Pastoral Neolithic sites on the southern Mbulu Plateau, Tanzania. Azania Archaeol Res Afr. 2013;48: 498–520. doi:10.1080/0067270X.2013.841927

24. Miller JM, Willoughby PR. Radiometrically dated ostrich eggshell beads from the Middle and Later Stone Age of Magubike Rockshelter, southern Tanzania. J Hum Evol. 2014;74: 118–122. doi:10.1016/j.jhevol.2013.12.011

25. Miller JM, Sawchuk EA, Reedman ALR, Willoughby PR. Land snail shell beads in the sub-Saharan archaeological record: When, where, and why? Afr Archaeol Rev. 2018;35: 347–378. doi:10.1007/s10437-018-9305-3

26. Werner JJ, Willoughby PR. Middle Stone Age technology and cultural evolution at Magubike Rockshelter, southern Tanzania. Afr Archaeol Rev. 2017;34: 249–273. doi:10.1007/s10437-017-9254-2

27. Biittner KM, Sawchuk EA, Miller JM, Werner JJ, Bushozi PM, Willoughby PR. Excavations at Mlambalasi Rockshelter: A terminal Pleistocene to recent Iron Age record in southern Tanzania. Afr Archaeol Rev. 2017;34: 275–295. doi:10.1007/s10437-017-9253-3

28. Miller JM. The ostrich eggshell beads of Mlambalasi Rockshelter, southern Tanzania. MA, University of Alberta. 2012. doi:10.7939/R3BT07

29. Gliganic LA, Jacobs Z, Roberts RG, Domínguez-Rodrigo M, Mabulla AZP. New ages for Middle and Later Stone Age deposits at Mumba Rockshelter, Tanzania: Optically Stimulated Luminescence dating of quartz and feldspar grains. J Hum Evol. 2012;62: 533–547. doi:10.1016/j.jhevol.2012.02.004

30. Prendergast ME, Luque L, Dominguez-Rodrigo M, Diez-Martin F, Mabulla AZP, Barba R. New excavations at Mumba Rockshelter, Tanzania. J Afr Archaeol. 2007;5: 217–44. doi:10.3213/1612-1651-10093

31. Mehlman MJ. Later Quaternary archaeological sequences in northern Tanzania. PhD, University of Illinois. 1989. Available: hdl.handle.net/2142/22520

32. Tryon CA, Lewis JE, Ranhorn KL, Kwekason A, Alex B, Laird MF, et al. Middle and Later Stone Age chronology of Kisese II Rockshelter (UNESCO World Heritage Kondoa Rock-Art Sites), Tanzania. PLoS ONE. 2018;13: e0192029. doi:10.1371/journal.pone.0192029
